# The Long-Term Impact of Cardiac Rehabilitation on Cognitive Function in Older Patients With Myocardial Infarction: A Systematic Review

**DOI:** 10.7759/cureus.67913

**Published:** 2024-08-27

**Authors:** Meaza Zergaw, Mohamed Elgendy, Alvin Billey, Asra Saleem, Bushra Zeeshan, Gayanthi Dissanayake, Sondos Nassar

**Affiliations:** 1 Internal Medicine, California Institute of Behavioral Neurosciences & Psychology, Fairfield, USA; 2 Orthopedics, California Institute of Behavioral Neurosciences & Psychology, Fairfield, USA; 3 Dermatology, California Institute of Behavioral Neurosciences & Psychology, Fairfield, USA; 4 Internal Medicine and Family Medicine, California Institute of Behavioral Neurosciences & Psychology, Fairfield, USA; 5 Medicine and Surgery, California Institute of Behavioral Neurosciences & Psychology, Fairfield, USA

**Keywords:** myocardial infarction, ischemic heart disease, myocardial ischemia, cognitive dysfunction, cognitive improvement, cognitive function, cardiac reconditioning, cardiovascular rehabilitation, cardiac rehabilitation

## Abstract

Myocardial Infarction (MI) is an obstruction in the coronary arteries, resulting in restricted blood flow and oxygen supply to the heart, leading to damage to the heart's tissues. Beyond the cardiovascular system, the impact of MI extends to potentially affecting cognitive abilities, especially in elderly populations. To optimize patient recovery and long-term outcomes, timely cardiac interventions and subsequent rehabilitation programs are essential. This systematic review aims to assess the potential benefits of cardiac rehabilitation (CR) in enhancing cognitive function among elderly individuals who have experienced an MI. The review adheres to the Preferred Reporting Items for Systematic Reviews and Meta-Analyses 2020 guidelines and utilizes PubMed, PubMed Central, Cochrane, Google Scholar, and ScienceDirect databases. Studies included in the review encompass meta-analyses, controlled trials, systematic/narrative reviews, randomized/nonrandomized trials, observational studies, and research articles published within the past five years. Only accessible, full-text English-language studies meeting the inclusion criteria are selected, while books, documents over five years old, animal studies, and individuals under 65 are excluded. Following a predefined template, the initial search identifies 4,915 studies. From this pool, 27 free full-text articles are then selected for quality appraisal based on relevance. After performing a quality assessment on each survey, 12 high-quality studies are included in this systematic review. The research studies demonstrate notable cognitive improvements among elderly patients who have experienced an MI and participated in CR programs. Additional clinical trial studies are recommended to substantiate these findings further and advance our understanding.

## Introduction and background

Myocardial infarction (MI), commonly known as a heart attack, occurs when plaque buildup in the arteries restricts blood flow to the heart, resulting in damage to the heart muscles due to a lack of oxygen supply [1]. The elderly population, defined as individuals aged 75 years and above, is increasingly becoming a significant percentage of patients presenting with acute myocardial infarction (AMI) [2]. While adults aged 75 and older make up only 6% of the United States population, they account for a substantial portion of AMIs, AMI-related deaths, and overall cardiovascular deaths in the country [3]. Older adults with MI tend to have more chronic health conditions and physical and cognitive impairments compared to younger individuals, which can contribute to their higher mortality risk [4].

The complications of MI are highly significant as they pose huge impacts on the quality of patients' lives. Research has shown that, in addition to long-term cardiac impairment, patients may also experience accelerated cognitive decline following MI. Changes during MI may contribute to a long-term process that affects cognitive function. Among the contributing factors, a sustained reduction in cerebral blood flow following cardiac dysfunction plays a crucial role [5]. This makes cognitive decline to be a significant health concern seen in elderly individuals after experiencing MI [6]. Therefore, timely detection of individuals with cognitive decline is essential, as the early identification and subsequent customization of treatment approaches can potentially enhance patient outcomes and guide prognosis [7].

Patients experiencing MI are typically managed with a combination of therapies. Early reperfusion treatments like percutaneous coronary intervention (PCI) are crucial, as they help prevent the development of cardiogenic shock and quickly restore left ventricular function. Pharmacological interventions, such as antiplatelet and anticoagulant medications, improve clinical outcomes. Additionally, optimal secondary prevention measures, including lifestyle changes, ongoing medical management, and comprehensive rehabilitation programs, are vital in enhancing long-term cardiac and cognitive performance [8]. Cardiac rehabilitation (CR) initiatives especially have proven to be highly effective for older adults living with cardiovascular disease (CVD). These programs are beneficial in addressing the unique and complex challenges faced by geriatric patients as they employ a variety of interventions, including exercise training, behavioral modification, education, and psychological counseling. These multifaceted efforts work to optimize outcomes and functionality for individuals with cardiovascular conditions [9]. However, elderly patients, especially those with cognitive impairments, are generally less inclined to participate in CR programs compared to their younger counterparts. As such, healthcare providers likely need to strengthen the continuity of care and improve accessibility to these programs for elderly patients after discharge from hospital care and transition to local health centers [10].

Limited research exists regarding cardiovascular interventions and clinical outcomes among older MI patients with cognitive impairment [11]. Therefore, the purpose of this systematic review is to further evaluate the long-term impact of interventions like CR on improving cognitive functioning in older patients who have experienced an MI.

## Review

Methods

The Population, Intervention, Comparison, and Outcome framework formulated the research question. This systematic review adhered to the Preferred Reporting Items for Systematic Reviews and Meta-Analyses (PRISMA) 2020 guidelines [12]. The inclusion criteria for this review encompassed meta-analyses, systematic reviews, traditional reviews, randomized clinical trial studies, case-control studies, cohort studies, research journals, and case reports, focusing on publications from the last five years. Conversely, the exclusion criteria eliminated papers published more than five years ago, animal studies, non-English publications, and individuals younger than 65.

Databases and Search Strategy

This systematic review, conducted in April 2024, involved an electronic search across various databases, including PubMed, Cochrane Library, and ScienceDirect. The search terms utilized encompassed Myocardial Infarction, Myocardial Ischemia, Ischemic Heart Disease, Cognitive Improvement, Cognitive Dysfunction, Cardiac Rehabilitation, Cardiovascular Rehabilitation, and Cardiac Reconditioning. The search strategy primarily employed the MESH approach in PubMed. A comprehensive summary of the databases and search methods is shown in Table 1.

**Table 1:** Summary of the databases and search methods PMC: PubMed Central

Databases	Keywords	Search strategy	Number of articles before filters	Inclusion criteria	Exclusion criteria	Search results
PubMed	Cardiac Rehabilitation, Cardiovascular Rehabilitation, Cardiac Reconditioning, Cognitive Function, Cognitive Improvement, Cognitive Dysfunction, Myocardial Infarction, Myocardial Ischemia, Ischemic Heart Disease	Cardiac Rehabilitation OR Cardiovascular Rehabilitation OR Cardiac Reconditioning OR ("Cardiac Rehabilitation/adverse effects"[Majr] OR"Cardiac Rehabilitation/methods"[Majr] OR "Cardiac Rehabilitation/mortality"[Majr]) AND Cognitive Function OR Cognitive Improvement OR Cognitive Dysfunction OR ("Cognition/classification"[Majr] OR "Cognition/drug effects"[Majr] OR "Cognition/physiology"[Majr]) AND Myocardial Infarction OR Myocardial Ischemia OR Ischemic Heart Disease OR ("Myocardial Ischemia/complications"[Majr] OR "Myocardial Ischemia/mortality"[Majr] OR "Myocardial Ischemia/rehabilitation"[Majr] OR "Myocardial Ischemia/therapy"[Majr])	580,281	English language only, age >65 years, associated data, studies between 2020 and 2024, free full-text human only, all types of articles	Age <65 years, animal studies, studies before 2020	2,129
Google Scholar	Cardiac Rehabilitation, Cardiovascular Rehabilitation, Cardiac Reconditioning, Cognitive Function, Cognitive Improvement, Cognitive Dysfunction, Myocardial Infarction, Myocardial Ischemia, Ischemic Heart Disease	"Cardiac Rehabilitation" OR "Cardiovascular Rehabilitation" OR "Cardiac Reconditioning" AND "Cognitive Function" OR "Cognitive Improvement" OR "Cognitive Dysfunction" AND "Myocardial Infarction" OR "Myocardial Ischemia" OR "Ischemic Heart Disease"	862	Studies between 2020 and 2024	Any study before 2020	221
ScienceDirect	Cardiac Rehabilitation, Cognitive Function, Myocardial Ischemia	Cardiac Rehabilitation AND Cognitive Function AND Myocardial Ischemia	2,122	Studies between 2020 and 2024, all articles type, all subject area, all publication title, only in English, open access, and open archive	Any study before 2020, studies other than English, and no open access	173
Cochrane Library	Cardiac Rehabilitation, Cardiovascular Rehabilitation, Cardiac Reconditioning, Cognitive Function, Cognitive Improvement, Cognitive Dysfunction, Myocardial Infarction, Myocardial Ischemia, Ischemic Heart Disease	"Cardiac Rehabilitation" OR "Cardiovascular Rehabilitation" OR "Cardiac Reconditioning" AND "Cognitive Function" OR "Cognitive Improvement" OR "Cognitive Dysfunction" AND "Myocardial Infarction" OR "Myocardial Ischemia" OR "Ischemic Heart Disease"	208	Last five years	Any studies before the last five years	46
PMC	Cardiac Rehabilitation, Cognitive Function, Myocardial Ischemia	Cardiac Rehabilitation AND Cognitive Function AND Myocardial Ischemia	7,708	Studies between 2020 and 2024, open access, and associated data	Studies before 2020	2,346

Results

Study Selection and Quality Assessment

The initial database search yielded 4,915 publications. From this, 200 articles were automatically deleted from Google Scholar. The remaining articles were imported into endnotes and sorted alphabetically. Subsequently, 65 duplicate articles were removed, both automatically and manually. The remaining articles were screened based on title and abstract, and 32 articles were sought for retrieval. Finally, 27 full-text articles were retrieved for quality evaluation based on their relevance to the research topic. Two independent reviewers screened the titles and abstracts of the unique records to determine eligibility using prespecified criteria. The same reviewers then independently assessed the full articles for quality and risk of bias, employing appropriate tools for the specific study type: Joanna Briggs Institute (JBI) Critical Appraisal Checklist for nonrandomized clinical trials, Appraisal Tool for Cross-Sectional Studies (AXIS) for cross-sectional studies, Cochrane Collaboration Risk of Bias Tool (CCRBT) for randomized clinical trials, Newcastle-Ottawa Scale (NOS) for cohort studies, Assessment of Multiple Systematic Reviews 2 for systematic reviews and meta-analyses, and Scale for the Assessment of Narrative Review Articles 2 (SANRA 2) for narrative reviews [13-18]. Any disagreements were resolved by discussion.

Each assessment tool had its criteria and different scoring. A point was awarded when a tool scored "LOW," "YES," "1," and "2." A score of at least 70% for each assessment tool was accepted. Following the quality evaluation, 12 high-quality studies were included in the review, consisting of three randomized controlled trials, three cohort studies, one nonrandomized clinical trial, two narrative reviews, and three cross-sectional studies. Of all the three high-quality cohort studies, one did not fully meet the age criteria. However, we decided to include it in the discussion because it was relevant and applicable across all age groups.

**Figure 1:**
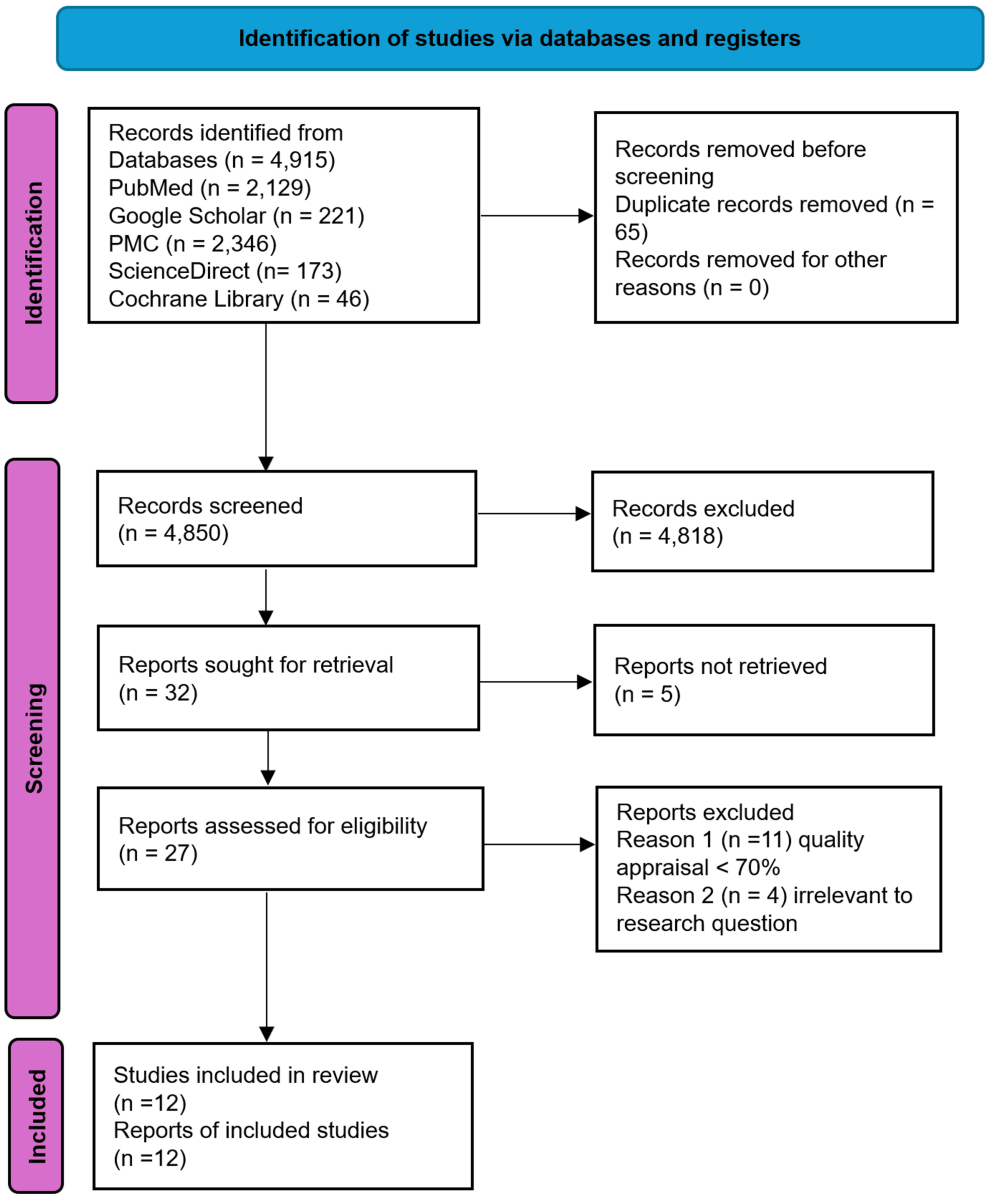
The PRISMA chart illustrating the screening process used to ultimately select the studies that were included in this systematic review PMC: PubMed Central; PRISMA: Preferred Reporting Items for Systematic Reviews and Meta-Analyses

A summary of the study selection process using the PRISMA flow diagram can be found in Figure 1.

Studies' Characteristics

Table 2 shows the characteristics of the studies included in the discussion part.

**Table 2:** Studies' characteristics SANRA 2: Scale for the Assessment of Narrative Review Articles 2; CR: cardiac rehabilitation; AXIS: Appraisal tool for Cross-Sectional Studies; MI: myocardial infarction; CCRBT: Cochrane Collaboration Risk-of-Bias Tool; NOS: Newcastle-Ottawa Scale; JBI: Joanna Briggs Institute; AMI: acute myocardial infarction; PCI: percutaneous coronary intervention; CVD: cardiovascular disease; PEKE: progressive exercise of kinetic energy; ACS: acute coronary syndrome; CI: cognitive impairment

Study	Study design	Quality assessment tool used	Study title	Age group	Data collection period	Study conclusion
Lutz and Forman [9]	Narrative reviews	SANRA 2	Cardiac rehabilitation in older adults: apropos yet significantly underutilized	Review summarizes the current data available regarding CR in older adults	Review summarizes the current data available regarding CR in older adults	Cardiac rehabilitation is effective for older adults with cardiovascular disease and can help address the specific complexities in this population
Liljeroos et al. [10]	Cross-sectional studies	AXIS	Self-perceived cognitive status and cognitive challenges associated with cardiac rehabilitation management: experiences of elderly myocardial infarction patients	≥65 years	Data were collected between 6 and 12-weeks after MI	Personalized interventions and better healthcare access can help overcome cognitive issues in cardiac rehab
Wagner-Skacel et al. [19]	Randomized controlled trials	CCRBT	The impact of cardiovascular rehabilitation on psychophysiological stress, personality and tryptophan metabolism: a randomized pilot feasibility study	40-80 years	October-November 2019	Multifaceted cardiac rehabilitation can enhance cardiac patients' psychological and physiological well-being during their initial recovery period
Asai et al. 2023 [20]	Cohort study	NOS	The impact of cardiac rehabilitation for older adults with heart failure who underwent invasive cardiac treatment eligible for long-term care needs certification: a retrospective cohort study	≥65 years	April 2014-March 2015	The study underscored the importance of cardiac rehabilitation for older adults with heart failure who require long-term care certification, in the context of an aging society
Huang [21]	Nonrandomized clinical trials	JBI Critical Appraisal Checklist	Effect of new cardiac rehabilitation mode on cardiac function, mental state and quality of life of postoperative patients with acute myocardial infarction treated with atorvastatin calcium tablet	55.21 ± 6.20 years	January 2018-January 2019	The new cardiac rehabilitation effectively improves cardiac function, mitigate negative emotions, and reduce complications in AMI after PCI, when combined with atorvastatin calcium tablets
Yang et al. [22]	Cohort study	NOS	Effect of cardiac rehabilitation care after coronary intervention on cardiac function recovery and negative mood in patients with myocardial infarction	36.26 ± 9.88 years	June 2022 to July 2023	Cardiac rehabilitation employs various interventions to address the multifaceted needs of patients, improve cardiac function and physical fitness, and support mental health recovery
Fujiyoshi et al. [23]	Cohort Studies	NOS	Effect of cardiac rehabilitation on cognitive function in elderly patients with cardiovascular diseases	≥70 years old	October 2015-June 2016	Cardiac rehabilitation can improve cognitive function in elderly CVD patients, shedding light on its efficacy for secondary prevention
Gaalema et al. [24]	Narrative reviews	SANRA 2	Cognition and exercise: general overview and implications for cardiac rehabilitation	Review summarizes the implications for cardiac rehabilitation within populations at high-risk for cognitive deficits	Review summarizes the implications for cardiac rehabilitation within populations at high-risk for cognitive deficits	CR programs should identify patients with cognitive challenges, provide tailored support, and encourage moderate-to-high intensity exercise to optimize cognitive health outcomes. Maintaining long-term exercise is crucial for sustaining cognitive gains after CR
Jiang et al. [25]	Randomized controlled trials	CCRBT	Effect analysis of kinetic energy progressive exercise in patients with acute myocardial infarction (AMI) after percutaneous coronary intervention (PCI): a randomized trial	59.62 ± 8.98 years	April 2019-April 2020	When PEKE was applied to patients with AMI after PCI, it was shown to effectively reduce adverse effects, and improve motor function, cardiac function, and their quality of life
Trubnikova et al. [26]	Randomized controlled trials	CCRBT	Beneficial effects of a short course of physical prehabilitation on neurophysiological functioning and neurovascular biomarkers in patients undergoing coronary artery bypass grafting	45-70 years	Patients are followed 7-10 days before and after coronary artery bypass graft surgery	Preoperative physical therapy improves early postoperative brain function, cognition, and neurovascular markers in cardiac surgery patients, suggesting it can enhance brain resilience and reduce cognitive decline
Gallagher et al. [27]	Cross-sectional studies	AXIS	Cognitive impairment and psychological state in acute coronary syndrome (ACS) patients: a prospective descriptive study at cardiac rehabilitation entry, completion and follow-up	66.2 (±8.22) years	Data collections were done in 2017	Mild CI is prevalent in post-ACS patients in cardiac rehab, impacting secondary prevention. CI often improves during/after rehab, but reasons are unclear, could be rehab itself or factors like psychology
Dong et al. [28]	Cross-sectional studies	AXIS	Associations of physical activity with cognitive function and daily physical function among Chinese individuals with heart disease: a cross-sectional study	65.26 (±9.85)	Data collections were done in 2015	There is observed benefits of physical activity and cognitive function

Qualities of Included Studies

Table 3 shows how randomized clinical trials were assessed using the CCRBT. Studies with a high risk of bias receive a "High" rating, studies with a low risk of bias receive a "Low" rating, and studies with an unknown risk of bias receive an "Unclear" rating.

**Table 3:** Quality assessment summary of randomized controlled trials using CCRBT CCRBT: Cochrane Collaboration Risk-of-Bias Tool

Study	Random sequence generation	Allocation concealment	Selective reporting	Other sources of bias	Performance bias	Detection bias	Attrition bias	Scoring
Wagner-Skacel et al. [19]	Low	Low	Low	Low	High	Low	Low	87.5%
Jiang et al. [25]	Low	Low	Low	Low	Unclear	Unclear	Low	71.42%
Trubnikova et al. [26]	Low	Low	Low	Low	Low	Low	Low	100%

Table 4 presents the assessment of all cohort studies using the NOS tool. The high-quality cohort studies scored "1" or "2" on each question, while the low-quality studies received a score of "0."

**Table 4:** Quality assessment summary of cohort studies using NOS Q: Question; NOS: Newcastle-Ottawa Scale

Study	Selection				Comparability	Outcome			Scoring
	Q1	Q2	Q3	Q4	Q1	Q1	Q2	Q3	
Asai et al. [20]	1	1	1	1	2	1	1	1	100%
Yang et al. [22]	1	1	1	1	1	1	1	1	100%
Fujiyoshi et al. [23]	1	1	1	1	1	1	1	1	100%

Table 5 shows the scoring of narrative reviews using the SANRA 2 checklist, which has six items. The high-quality reviews scored "1" or "2" on each item, while the low-quality reviews received a score of "0."

**Table 5:** Quality assessment summary of narrative reviews using the SANRA 2 SANRA 2: Scale for the Assessment of Narrative Review Articles 2

Study	Justification of the article's importance for the readership	Statement of concrete aims or formulation of questions	Description of the literature search	Referencing	Scientific reasoning	Appropriate presentation of data	Scoring
Lutz and Forman [9]	2	2	0	2	2	2	83.33%
Gaalema et al. [24]	2	2	0	2	2	2	83.33%

Table 6 displays the scoring of cross-sectional studies using the AXIS with 20 assessment questions. The accepted high-quality studies have "Yes" answers for all questions in the tool, except for questions 13 and 19, where a "No" answer also indicates high quality. Low-quality studies are identified by "No" answers, except for questions 13 and 19, where a "Yes" answer also suggests low quality. The answer "Don't know" is provided when the quality is unclear.

**Table 6:** Quality assessment summary of cross-sectional studies using AXIS Q: question; Y: yes; N: no; DK: don't know; AXIS: Appraisal tool for Cross-Sectional Studies

Study	Introduction	Methods										Result					Discussion		Others		Scoring
	Q1	Q2	Q3	Q4	Q5	Q6	Q7	Q8	Q9	Q10	Q11	Q12	Q13	Q14	Q15	Q16	Q17	Q18	Q19	Q20	
Liljeroos et al. [10]	Y	Y	Y	Y	Y	Y	Y	Y	Y	Y	Y	Y	N	Y	Y	Y	Y	Y	Y	Y	95%
Gallagher et al. [27]	Y	Y	Y	Y	Y	Y	Y	Y	Y	Y	Y	Y	N	Y	Y	Y	Y	Y	Y	Y	95%
Dong et al. [28]	Y	Y	Y	Y	Y	Y	DK	Y	Y	Y	Y	Y	DK	DK	Y	Y	Y	Y	Y	Y	80%

Table 7 presents the quality assessment of the nonrandomized clinical trials using the JBI Critical Appraisal Checklist. Questions with a "Yes" answer indicate high quality, while "No", "Unclear", and "Not Applicable" responses suggest low quality.

**Table 7:** Quality assessment summary of nonrandomized clinical trials using the JBI Critical Appraisal Checklist YQ: question; Y: yes; JBI: Joanna Briggs Institute

Study	Q1	Q2	Q3	Q 4	Q5	Q6	Q7	Q8	Q9	Scoring
Huang [21]	Y	Y	Y	Y	Y	Y	Y	Y	Y	100%

Discussion

Cardiac Rehabilitation

CR has emerged as a pivotal component in the management of CVD. Medical evidence suggests that this comprehensive program can significantly improve the health outcomes and prognosis for patients with various heart conditions. CR is a multifaceted approach encompassing various fields, including physical training, medication management, psychological interventions, and nutritional guidance. Through structured exercise programs such as aerobic exercise, strength training, yoga, and rehabilitation gymnastics, CR aims to enhance physical activity levels, increase exercise endurance, and improve cardiopulmonary function and quality of life. Concurrently, rehabilitation care can provide targeted drug treatment for patients recovering from MI. Moreover, CR employs psychological support and cognitive behavioral therapy to help patients cope with emotional challenges, effectively reducing symptoms of depression and anxiety.

Regarding nutritional guidance, rehabilitation care ensures patients adopt appropriate diets to maintain heart health and physical function. CR nursing has achieved comprehensive results through the collaborative efforts of a multidisciplinary team. This team, typically comprised of cardiologists, physicians, and other healthcare professionals, develops a comprehensive treatment plan and delivers a full range of physical, psychological, and social interventions to optimize the rehabilitation effect and improve patients' overall quality of life [19-22].

The importance of CR lies in its ability to facilitate the patient's return to an active and independent lifestyle. Through a detailed assessment of the patient's physical function, clinical status, and psychological well-being, a personalized rehabilitation plan is developed to ensure the relevance and effectiveness of the treatment. This holistic approach aims to address the diverse aspects of the patient's recovery, including physical, medical, and psychosocial domains [22]. CR has been particularly beneficial in the critical period following acute cardiovascular events, such as MI or coronary artery bypass surgery (CABG). During this time, the supervised and structured reintroduction to physical activity can be invaluable, helping patients overcome significant physiological and psychological barriers.

**Figure 2:**
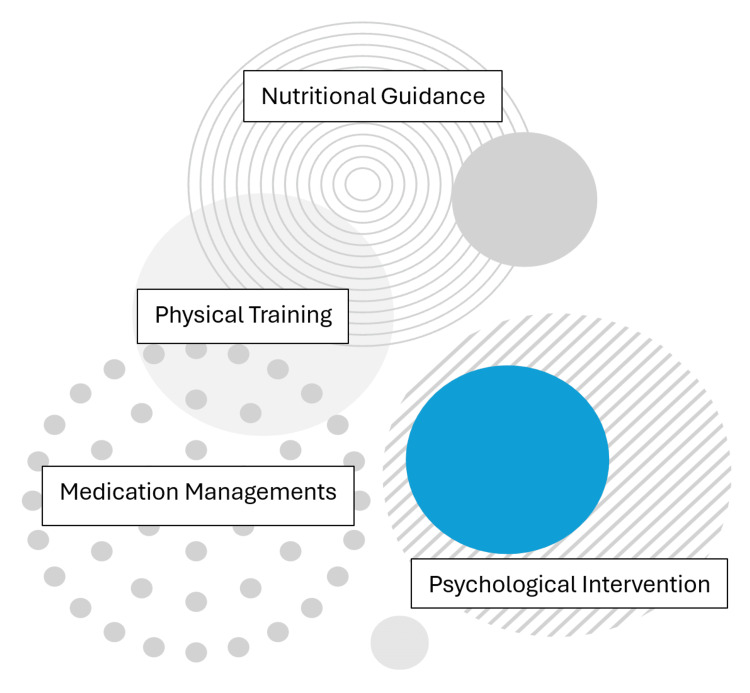
CR components CR: cardiac rehabilitation Image credit: Meaza Zergaw

Moreover, as a disease management program, CR provides an opportunity for medication review, care coordination, and frequent evaluation of the patient's progress. Its eligibility has expanded over time, now including patients with a wide range of cardiovascular conditions, including stable coronary artery disease, recent PCI, heart failure with reduced ejection fraction, valvular heart disease, and peripheral arterial disease [9]. Furthermore, CR has emerged as a valuable preventive measure for elderly patients, helping to improve their functional capacity, physical activity levels, and ability to maintain an independent lifestyle while also reducing mortality. Overall, CR's comprehensive and personalized nature has made it an essential component in the continuum of care for patients with CVD, offering a multidimensional approach to optimize their outcomes and well-being [10]. Figure 2 shows the components of CR.

The Heart-Brain Continuum Hypothesis

Emerging evidence suggests a strong connection between older patients' cognitive decline and the start of CVD, a phenomenon known as the "Heart-Brain Continuum" hypothesis. According to this notion, thrombotic states, heart problems, and vascular endothelial dysfunction can all be brought on by the development or initiation of CVD, which can then negatively impact cognitive performance. On the other hand, the presence of cognitive impairment may exacerbate the condition of CVD through factors such as poor medication adherence, inadequate nutritional status, and limited physical activity. As a result, the preservation of cognitive function is a vital consideration for secondary prevention strategies, and it can serve as a valuable therapeutic and interventional target for elderly patients with CVD [23].

Numerous studies have demonstrated that various forms of physical exercise are associated with improvements across different cognitive domains, including attention, executive function, and global cognition. Exercise can be categorized into physical training, such as endurance and strength-based activities, and motor training, which includes balance, flexibility, and coordination exercises. When developing an exercise regimen, it is crucial to incorporate a variety of these exercises to target individual strengths and limitations. The specific exercise modalities can impact the types of cognitive benefits observed, with acute bouts enhancing attention, reaction time, and inhibition, while long-term engagement can help slow cognitive decline. The strongest evidence supports the benefits of endurance exercise or endurance and strength training combinations, with a clear dose-dependent relationship; greater exercise volume is linked to greater cognitive enhancements.

Studies have specifically examined the effects of exercise on cognition in individuals with CVD, often in the context of CR programs, consistently finding that increased exercise participation is associated with improvements in overall cognition and specific areas like attention, executive function, and memory. Moreover, it has been shown that participating in exercises like yoga improves the overall quality of sleep in patients with CR history. However, the available evidence does not indicate similar benefits for language-related cognitive abilities [19,24].

Researchers have proposed that multiple factors contribute to the cognitive improvements observed following CR interventions. One key factor is endothelial function, which can enhance cerebral microcirculation. This is achieved through several mechanisms, including increased nitric oxide and prostacyclin production and increased tissue plasminogen activator activity. Nitric oxide is a potent vasodilator that can improve blood flow to the brain, while prostacyclin can also enhance cerebral blood flow. Additionally, increased tissue plasminogen activator activity can improve the breakdown of blood clots, further enhancing perfusion. Improved shear stress on the blood vessel walls can also promote the release of vasodilating substances and improve microvascular function.

Furthermore, restoring cardiac function, as evidenced by reduced brain natriuretic peptide levels and improved left ventricular ejection fraction, may also contribute to improved cognitive function. Better cardiac performance can increase overall blood flow and perfusion to the brain, which is essential for cognitive processes. The improved cerebral perfusion may also promote synaptic plasticity, reinforcing cognitive processes like working memory and psychomotor speed. Moreover, CR programs have been shown to significantly improve frontal cortical function, although the underlying reasons for these specific improvements remain unclear. While increased whole cerebral blood flow has been reported after exercise therapy, the localized increase in cerebral flow within specific cortical regions following rehabilitation programs has not been conclusively demonstrated. The impact of these multifaceted mechanisms underscores the cognitive benefits associated with CR [23,24].

**Figure 3:**
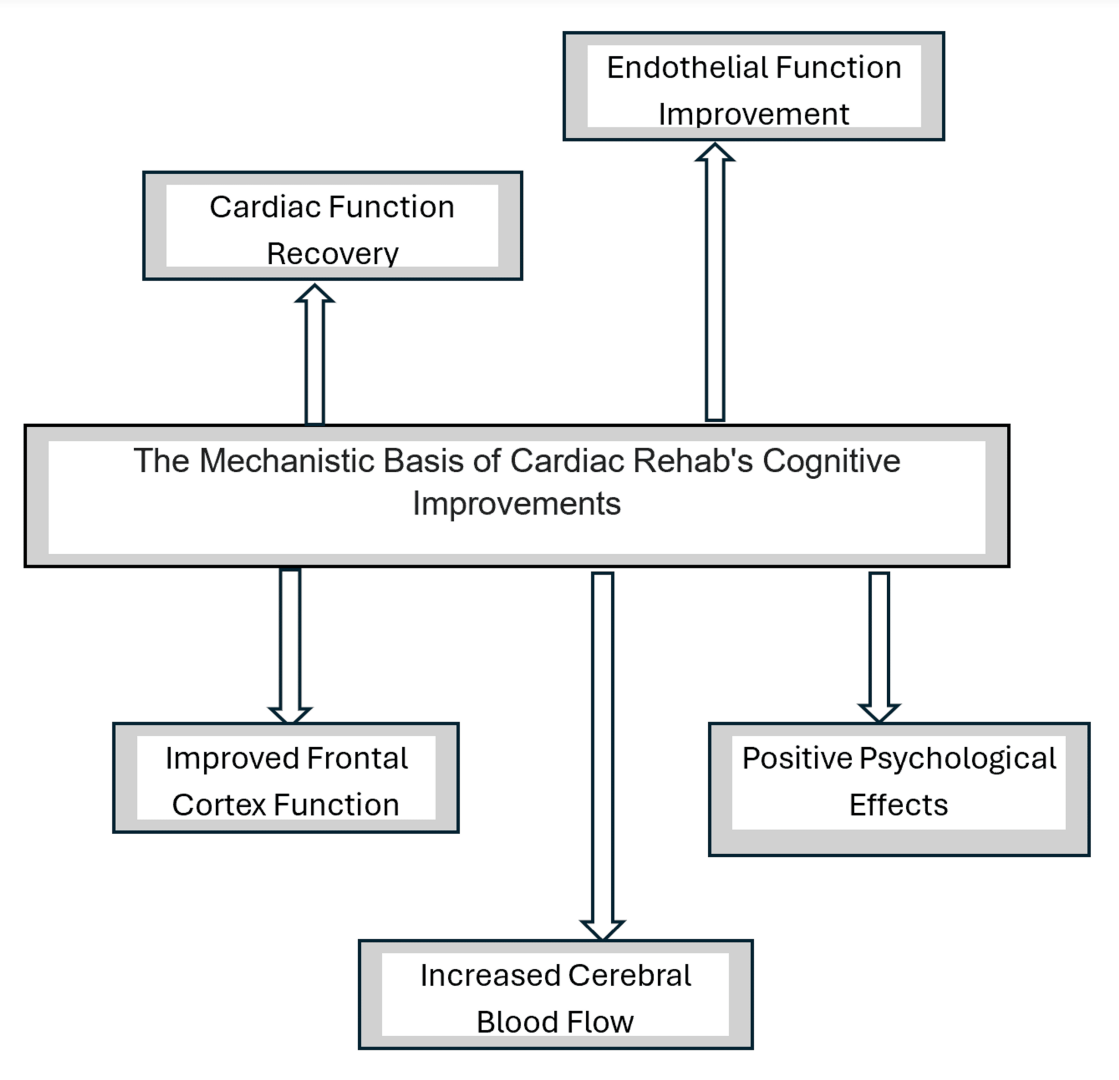
The mechanistic basis of cardiac rehab's cognitive improvements Image credit: Meaza Zergaw

It is also shown that the positive psychological effects of CR, such as increased activity, optimism, and improved exercise habits, may also contribute to the observed cognitive benefits, potentially through the elevation of dopamine levels. Dopamine is a neurotransmitter that is associated with improved cognitive function and mood, and the positive psychological effects of CR may lead to increased dopamine levels, further enhancing cognitive performance [21]. Figure 3 shows the mechanistic basis of cardiac rehab's cognitive improvements.

A randomized clinical trial conducted on patients who had experienced AMI and underwent PCI revealed that progressive exercise of kinetic energy (PEKE), a new type of rehabilitation approach, significantly improved symptoms, physical function, cognitive function, psychosocial function, and satisfaction scores compared to those who underwent standard cardiac intervention over six months. These findings indicated that PEKE effectively enhanced AMI patients' quality of life and prognosis post-PCI.

Researchers suggested that PEKE's benefits might be attributed to its ability to regulate vascular tension, reduce peripheral vascular resistance, and improve aortic compliance. These physiological effects were believed to alleviate micro vasospasm, normalize blood pressure, and ultimately enhance patients' exercise endurance, all crucial factors for optimizing quality of life. The trial underscored PEKE as a promising addition to rehabilitation strategies for AMI patients following PCI, demonstrating its potential to positively impact various aspects of patient health and well-being. The improvements observed in physical, cognitive, and psychosocial functions, alongside enhanced satisfaction scores, highlighted PEKE's comprehensive benefits in CR [25].

Another randomized clinical trial also demonstrated that postoperative physical therapy (PT) can reduce cognitive impairment in CBAG surgery patients following MI. However, the effectiveness of physical rehabilitation prior to cardiac surgery remains under investigation. Interestingly, patients who underwent PT 7-10 days before CABG surgery experienced lower slow-wave theta brain activity increases. This brain wave pattern is associated with perioperative brain damage, as well as mild cognitive impairment and dementia. The beneficial effects of PT on the brain may manifest as improved resilience to acute, global ischemia during CABG, as evidenced by the observed electrical activity.

Furthermore, study results suggest that serum biomarkers such as brain-derived neurotrophic factor (BDNF), S100β protein, and neuron-specific enolase (NSE) could be useful in evaluating the effectiveness of rehabilitation programs for CABG patients. For instance, a short aerobic exercise regimen was linked to increased presurgical BDNF levels, indicating that this neurotrophic factor may play a role in training-induced cognitive enhancement. However, BDNF changes may not be the only factor driving cognitive improvements. Notably, patients who underwent physical prehabilitation also had lower post-CABG serum concentrations of S100B and NSE, suggesting less brain damage in these individuals [26].

The Effects of CR on Cognitive Performance

Extensive research has revealed that mild cognitive impairment is a common occurrence among patients with acute coronary syndrome who participate in CR programs. However, this cognitive impairment appears to be transient, as evidenced by a significant decrease in its prevalence during the rehabilitation process and subsequent follow-up. The studies have demonstrated substantial improvements in various cognitive domains, including verbal attentional abilities and memory (such as verbal learning, visuospatial memory, and attention-executive-psychomotor functions), from the start of the rehabilitation program to discharge. The only exception to this overall trend of improvement was a nonsignificant increase in impairment in visual attention, a finding that warrants further exploration, particularly in relation to an individual's changes in cardiovascular health during the rehabilitation period. This suggests that the cognitive benefits of CR may extend beyond physical improvements, potentially aiding in the recovery and overall well-being of these patients. The insights gained from these studies can help healthcare providers develop more comprehensive and personalized rehabilitation programs, targeting both the physical and cognitive aspects of patient recovery [27].

The benefits of consistent exercise therapy within cardiovascular rehabilitation programs have been well-documented in mitigating cognitive decline. Healthcare professionals are advised to monitor patients' cognitive status to ensure adherence to medication and lifestyle modifications, which ultimately aids in managing disease progression and recurrence effectively [20]. Previous research has demonstrated the effectiveness of comprehensive CR programs that incorporate cognitive function training in maintaining and enhancing cognitive function. However, more recent studies have suggested that CR alone can also lead to improvements in cognitive function without the need for specific cognitive training [23].

Supervised exercise therapy is recommended, as different exercise intensities can affect cognitive function differently. Engaging in 30-119 minutes of moderate-to-low physical activity per day has been found to yield the most positive impact on cognition, while higher intensity activities such as vigorous, very vigorous, and maximal exercise may have a negative effect, as they can disrupt metabolism in the frontal cortex of the brain in patients with heart disease [28].

The significance of this systematic review lies in its ability to guide physicians in better understanding and implementing CR programs, which can lead to enhanced patient outcomes. It is a valuable resource for healthcare professionals, demonstrating the potential benefits of CR programs in improving the cognitive functioning of elderly patients with MI and emphasizing the importance of a holistic approach to postinfarction care. By highlighting the effectiveness of these rehabilitation programs, the review can help healthcare providers make informed decisions and tailor their interventions to better address the needs of this patient population, ultimately leading to improved quality of life and long-term outcomes. This systematic review recognizes that its scope was limited, as it only included freely available articles published within the past five years. The findings could have been further strengthened by incorporating data from inaccessible studies and remotely published articles, which would have provided a more comprehensive understanding of the topic.

## Conclusions

This comprehensive study aims to investigate the potential benefits of CR programs for elderly patients who have experienced an MI, with a specific focus on the impact of these programs on cognitive functioning. The findings suggest that CR programs can indeed lead to significant improvements in cognitive function, as they positively influence various factors, such as cardiac performance, cortical function, and psychological well-being. The study highlights the importance of incorporating a multifaceted approach to post-MI care, addressing both the physical and cognitive needs of these elderly patients. By addressing cognitive function, rehabilitation programs can potentially enhance patients' overall quality of life and independence, enabling them to manage their conditions better and participate in their care. To further strengthen the findings, the study recommends conducting additional clinical trials to expand the understanding of the relationship between CR and cognitive function in the elderly population. This would provide a more robust evidence base to support the incorporation of cognitive assessments and interventions into standard CR programs, ultimately leading to improved outcomes for elderly patients who have suffered MI.
